# The 90th anniversary of Andrija Štampar School of Public Health

**DOI:** 10.3325/cmj.2017.58.330

**Published:** 2017-10

**Authors:** Mirjana Kujundžić Tiljak

**Affiliations:** University of Zagreb, School of Medicine, Andrija Štampar School of Public Health, Zagreb, Croatia*; mkujundz@snz.hr*

This year *Andrija Štampar School of Public Health* celebrates its 90th anniversary. The School was opened on October 3, 1927 as a result of professor Andrija Štampar’s vision and many years of work. At the beginning of its activity, the School of Public Health was a joint organization with the Hygienic Institute. At that time, the School’s task was to study circumstances that could adversely affect public health and to educate people about major health issues. State-of-the-art technology was used, including several film workshops, which produced quite a number of educational films regarding public health.

In 1947, the School became part of the University of Zagreb School of Medicine. From its beginnings to the present day, the most important role of the School has been education of medical doctors at all levels in the range of fields, from outpatient health care to public health competencies. The School maintains postgraduate courses in hygiene and social medicine and primary health care. The first postgraduate study in public health took place in 1947.

Specialization in general practice or family medicine at the School started in 1960. It was the first such program globally. Nowadays, at the Andrija Štampar School of Public Health medical students continuously gain public health knowledge and skills within different courses. Postgraduate specialist studies in family medicine, school and adolescent medicine, occupational health and sports medicine, public health medicine, epidemiology, and health management are also provided at the School. In addition, the School contributes to postgraduate doctoral studies “Biomedicine and Health” at the University of Zagreb School of Medicine.

During its history, the inner structure of the School underwent changes several times. Departments and institutes were founded and closed, merged or separated. Today, within the School of Public Health, along with the Library “Andrija Štampar”, the Office of Educational Technology and the Secretariat, there are five departments of the School of Medicine including Department of Social Medicine and Health Care Organization, Department for Medical Statistics, Epidemiology and Medical Informatics, Department of Health Ecology, Occupational Health and Sports Medicine with the Laboratory for Water and Balneoclimatology Research, Department of Medical Sociology and Health Economics, and Department of Family Medicine.

The social, epidemiological, technological, and political circumstances over the course of 90 years have changed constantly. Today, almost nothing is the same as it was at the time of the School’s foundation. Immediate goals and tasks are necessarily also different, but the strategy that leads to health for all remains unchanged. From its foundation to the present day, the School has been an exceptional and recognizable project that set high goals for national health policy and medical profession. Many of these goals have been realized in a magnificent way. The School has changed the destiny of a large number of people positively. It began with health projects in Turopolje and continued in neighboring and remote countries and continents. We have partnered in projects with colleagues from many countries including Japan, China, the Netherlands, the United Kingdom, the United States, and others. Today, the School is part of the European and global network of public health education and research institutions. It actively cooperates with the Association of Schools of Public Health in the European Region (ASPHER) and the World Health Organization (WHO). The School includes two WHO Collaborating Centers, the one for HIV strategic information and the one for Occupational Health.

As long as illness and disease occur, as long as there are health problems in individuals and vulnerable population groups, our School of Public Health continues with its mission. Andrija Štampar, our founder, our great teacher and visionary, has set high educational goals for public health professionals. Previous goals of sanitation and fight against infectious diseases, malaria, and tuberculosis are today largely replaced by new challenges posed by cardiovascular diseases and addiction and mental health issues. Today, in the 21st century, the School’s task is to convey new insights through contemporary communication channels and technologies, supporting public health with best available scientific evidence and translational research, building the bridge between public health theory and practice. Working together, academic researchers and local communities bring us into a realistic world of needs and opportunities. By strengthening public health competency in local communities, we provide citizens with support in achieving better health. The translation circuit loop is closed by simultaneous application of accumulated knowledge, both in practice planning and management for health and in teaching at the graduate, specialist, and doctoral level.

Our main aim in the years to come is to sensitize young researchers and promote their inclusion into public health science to ensure better understanding and functioning of the health care system as a whole.

